# The diagnostic issues of Adult attention deficit hyperactivity disorder (ADHD): A systematic literature review

**DOI:** 10.1192/j.eurpsy.2023.2018

**Published:** 2023-07-19

**Authors:** F. Askri, A. Aissa, S. Jedda, Y. Zgueb, U. Ouali

**Affiliations:** Avicenna, razi psychiatric hospital, Manouba, Tunisia

## Abstract

**Introduction:**

Attention deficit hyperactivity disorder (ADHD) is a condition that is usually diagnosed in childhood . However studies have shown that there is a propotion of adults with ADHD that did not have the disorder in their early ages and have been diagnosed in adulthood . Symptoms of adult ADHD are often confused with other psychiatric conditions due to the similarities in clinical criteria . Diagnosing Adult ADHD is still challenging due to lack of gold standard instruments , which can interfere with the therapy .

**Objectives:**

Identify the assessement measures that improve the early diagnosis of Adult ADHD

**Methods:**

A systematic literature search was executed using the PUBMED and GOOGLEscholar databases from 2009 to 2022 exploring the different assessement tools of adult ADHD using the keywords : Adult ADHD – Diagnosis issues – Assessement .

**Results:**

Results show diagnosing Adult ADHD can be by using three different assessement methods : clinical interview – ADHD behaviour rating and neuropsychological tests .

Clinical interview has a high sensitivity but low speceficity in diagnosing ADHD in adults .

Harrison and Al showed the CAARS scale appears to have adequate specificity in a young adult population giving it has validity to identify invalid symptom presentation.

The TOVA and the Conners CPT are the two CPTs widely used in ADHD assessment.

It is not clear whether the TOVA or Conners CPT has better diagnostic accuracy .

Cognitive test batteries dont show specific results in identifying ADHD

**Conclusions:**

To sum up , diagnosing adult ADHD is a challenging situation due to the lack of specifity in the assessemnt tools . This work highlights the necessity of further studies to improve the diagnostic process and the ability to create a universal guidelines . That way , early interventions and treatments can take place and avoid the confusion with other conditions .

**Disclosure of Interest:**

None Declared

